# Mature and Myelinating Oligodendrocytes Are Specifically Vulnerable to Mild Fluid Percussion Injury in Mice

**DOI:** 10.1089/neur.2023.0037

**Published:** 2023-06-29

**Authors:** Alexandra A. Adams, Teresa L. Wood, Haesun A. Kim

**Affiliations:** ^1^ Department of Biological Sciences, Physiology, and Neuroscience, New Jersey Medical School, Rutgers University, Newark, New Jersey, USA; ^2^ Department of Pharmacology, Physiology, and Neuroscience, New Jersey Medical School, Rutgers University, Newark, New Jersey, USA

**Keywords:** BCAS1, CC1, corpus callosum, FluoroMyelin™, GST-π, mild TBI, oligodendrocytes

## Abstract

Myelin loss and oligodendrocyte death are well documented in patients with traumatic brain injury (TBI), as well as in experimental animal models after moderate-to-severe TBI. In comparison, mild TBI (mTBI) does not necessarily result in myelin loss or oligodendrocyte death, but causes structural alterations in the myelin. To gain more insight into the impact of mTBI on oligodendrocyte lineage in the adult brain, we subjected mice to mild lateral fluid percussion injury (mFPI) and characterized the early impact (1 and 3 days post-injury) on oligodendrocytes in the corpus callosum using multiple oligodendrocyte lineage markers (platelet-derived growth factor receptor [PDGFR]-α, glutathione *S*-transferase [GST]-π, CC1, breast carcinoma-amplified sequence 1 [BCAS1], myelin basic protein [MBP], myelin-associated glycoprotein [MAG], proteolipid protein [PLP], and FluoroMyelin™). Two regions of the corpus callosum in relation to the impact site were analyzed: areas near (focal) and anterior (distal) to the impact site. mFPI did not result in oligodendrocyte death in either the focal or distal corpus callosum, nor impact on oligodendrocyte precursors (PDGFR-α^+^) and GST-π^+^ oligodendrocyte numbers. In the focal but not distal corpus callosum, mFPI caused a decrease in CC1^+^ as well as BCAS1^+^ actively myelinating oligodendrocytes and reduced FluoroMyelin intensity without altering myelin protein expression (MBP, PLP, and MAG). Disruption in node-paranode organization and loss of Nav1.6^+^ nodes were observed in both the focal and distal regions, even in areas without obvious axonal damage. Altogether, our study shows regional differences in mature and myelinating oligodendrocyte in response to mFPI. Further, mFPI elicits a widespread impact on node-paranode organization that affects regions both close to and remotely located from the site of injury.

## Introduction

Myelin is a crucial component of the white matter tracts given that it ensures fast axonal conductivity and is vital for neuronal survival. Myelinated axons are more resistant to traumatic brain injury (TBI), underscoring the protective role that myelin provides axons against mechanical injury.^1^ Further, several studies have shown that loss of myelin after TBI exacerbates axonal damage by facilitating a post-traumatic influx of calcium into the axons.^2,3^ Therefore, it is important to better understand the mechanisms underlying myelin loss and oligodendrocyte pathology post-TBI.

Axonal degeneration and oligodendrocyte death are prevalent during the early phase of moderate-to-severe TBI, which consequently leads to myelin loss.^4–8^ In comparison, oligodendrocyte death or myelin loss is not apparent after mild TBI (mTBI).^9,10,12–14^ Studies have also reported myelin loss on intact axons or intact myelin in areas of axonal degeneration.^10,14^ Myelin remodeling by pre-existing oligodendrocytes and disruption in oligodendrocyte-to-axon interaction have also been reported after mTBI.^15^ We have recently shown that mechanical impact induces an oligodendrocyte autonomous event that modulates myelin protein maintenance without causing cell death.^16^ Therefore, mTBI-associated oligodendrocyte pathology may involve mechanisms that are independent of oligodendrocyte death or axonal loss.

Fluid percussion injury (FPI) generates widespread white matter injury; thus, it is an ideal experimental model to examine TBI-induced pathology in regions located remotely from the impact site.^17^ To better understand mTBI impact on oligodendrocytes, we undertook a study to define the early-stage oligodendrocyte response to mild FPI (mFPI) in the mouse brain. Specifically, we conducted a detailed examination using multiple oligodendrocyte lineage markers in the corpus callosum, in two different regions in relation to the site of injury: immediately inferior to (focal) and anterior to (distal) the impact site. Our results show that there are regional differences in oligodendrocyte response to mFPI, where the immediate impact is mostly observed on mature-to-myelinating oligodendrocytes close to the site of injury. In regions located remotely, mFPI impacts the functional component of myelinated fiber on mostly intact axons without a direct impact on the oligodendrocytes.

## Methods

### Lateral mild fluid percussion injury

All male mice (C57BL/6) were used between 3 and 4 months of age. Detailed surgical procedure and the number of mice used are described in the Supplementary Materials and Methods. mFPI was defined by a righting reflex time of 70–270 sec (2.450 ± 0.988 min), as described previously.^18^ Injury righting times correlated to pressure pulses of 1.22–1.42 atm (1.33 ± 06 atm or 19.48 ± 0.89 psi). Sham-injured animals underwent the same experimental procedures, but were not subjected to the injury-inducing pressure pulse (righting times 0.34 ± 0.13 min). A brief period (<1 min) of apnea was typically observed in mFPI animals, which was resolved with no or minimal intervention. Only a small percentage of mFPI animals experienced mortality attributable to an inability to resume normal breathing after apnea (<5%).

### Antibodies and immunohistochemistry

Frozen brain sections (14 μm) were prepared for immunohistochemistry within the focal (interaural positions 4.04–3.56 mm and bregma positions 0.25–0.23 mm) and distal (interaural positions 4.40–4.89 mm and bregma positions 1.09–0.61 mm) corpus callosum. All the immunostaining procedures and antibody information are described in the Supplementary Materials and Methods. For Prussian blue and FluoroMyelin™ staining, the manufacturer protocols were followed (Sigma-Aldrich, St. Louis, MO; Thermo Fisher Scientific, Waltham, MA). Images were captured using either a Carl Zeiss MicroImaging LSM 510NLD Meta laser scanning multi-photon confocal microscope (Carl Zeiss AG, Oberkochen, Germany) or a Nikon Eclipse TE2000-U epifluorescence microscope (Nikon Corporation, Tokyo, Japan).

### Quantification of immunohistochemistry

All analyses were performed using Fiji open-source software. Only cells residing within the corpus callosum were quantified in all analyses. Imaging parameters for all samples included in each data set were maintained across samples. For all analyses, at least two images from two non-serial sections were quantified. Beta-amyloid precursor protein (βAPP)^+^ axons and FluoroMyelin intensity were quantified as a percentage of total area only within the corpus callosum. All images from sections immunolabeled for βAPP or FluoroMyelin were acquired using the same intensity settings. Total number of nodes was calculated using the intensity thresholding and particle analysis functions in Fiji. Intensity threshold and particle size were set uniformly for all images used in the analysis. Particle analysis was used to measure the total number of nodes that fell within the defined size and intensity threshold settings. Any particles out of the size or intensity range were not counted.

### Sodium dodecyl sulfate polyacrylamide gel electrophoresis and western blot analysis

Sham and injured brains were collected from CO_2_ euthanized animals at 3 days post-injury (DPI). Then, 1-mm coronal slices of the corpus callosum in the distal and focal regions were collected using mouse acrylic brain matrices and lysed in Nonidet P-40 (NP-40) lysis buffer (1% NP-40, 1% glycerol, 2.5 mM of ethylene glycol tetraacetic acid, 2 mM of ethylenediaminetetraacetic acid, 1 mM of sodium orthovanadate, 1 mM of phenylmethylsulphonyl fluoride, 10 mM of sodium fluoride, 10 μg/uL of aprotinin, and 20 μM of leupeptin). Tissue was homogenized in lysis buffer using a Dounce homogenizer on ice. Gel electrophoresis and protein detection were performed as described previously.^19^

### Statistical analysis

Statistical analyses were performed using GraphPad Prism Software (version 8; GraphPad Software Inc., La Jolla, CA). Two-way analysis of variance with Tukey's *post hoc* analysis was used to determine statistical significance in comparisons of multiple groups, and standard *t*-test in comparisons of only two groups. *p* < 0.05 was considered significant.

## Results

### Characterization of mild fluid percussion injury in mice

**FIG. 1.**
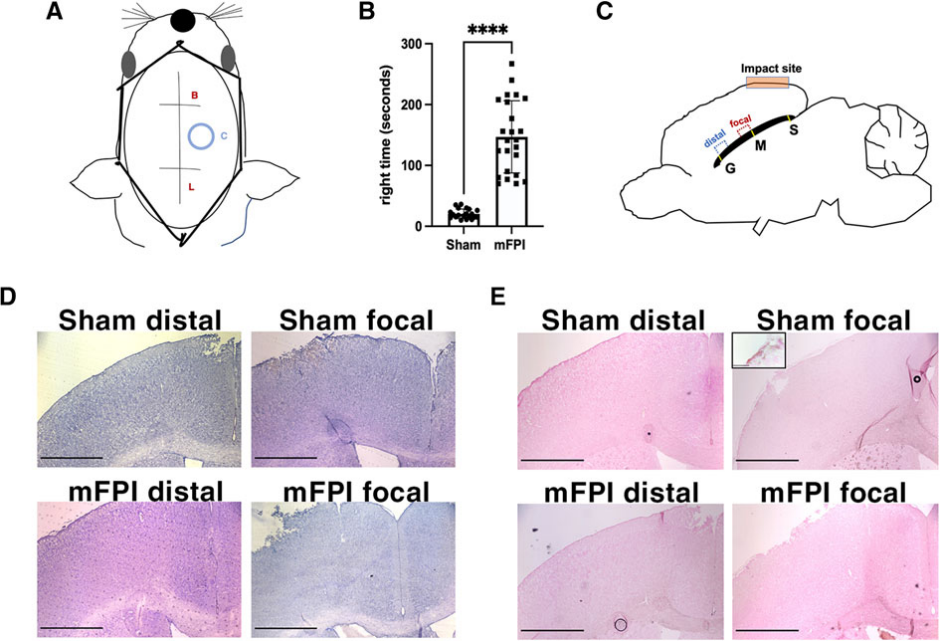
Overview of the injury system and anatomical regions of interest. (**A**) Schematic of the location of the craniectomy site from a superior view. Lines indicate the sagittal sutures, where “B” indicates bregma, “L” lambda, and “C” the craniectomy site. (**B**) Graphical representation of the righting time of all sham and injured animals included in the study (*p* < 0.0001). *N* = 21 sham and 25 mFPI mice (each data point reflects 1 animal). (**C**) Schematic of brain regions and location of the injury hub or “impact site,” where the corpus callosum is denoted in black, “S” indicates splenium, “M” midbody,” and “G” genu. (**D**) Hematoxylin-eosin stain of the tissue cytoarchitecture of the cortex and corpus callosum of sham and injured mice. (**E**) Prussian blue staining was used to determine whether blood extravasation occurred as a result of mFPI. Both sham and mFPI animals experienced superficial bleeding in the focal cortex at the craniectomy site that was observed only rarely in deeper layers of the cortex or corpus callosum. Hemosiderin deposits were not observed in the distal cortex or corpus callosum in either case. Inset in panel E demonstrates an example of superficial bleeding level of the cortex in a sham-injured animal, as indicated by the red/ brown deposits. Scale bars in all images = 500 μm. mFPI, mild fluid percussion injury .

For mFPI animals, the averaged peak pressure pulse at injury induction was 19.48 ± 0.89 psi (1.325 ± 0.060). mFPI animals experienced a significantly increased righting time compared to sham animals (Fig. 1B). Focally injured and distal regions of the corpus callosum were analyzed separately. Importantly, the distal region only included the area of the corpus callosum positioned entirely anterior to the site of the craniectomy (Fig. 1A, C).

We also defined mTBI as an injury without hemorrhaging or severe tissue loss, which are characteristics associated more frequently with moderate-to-severe TBI.

To monitor hemorrhaging and cortical tissue loss, we used Prussian blue and hematoxylin-eosin (H&E) staining, respectively. H&E staining showed largely intact tissue cytoarchitecture in sham and injured mouse brains, both in the focal and distal regions. Both sham and mFPI brains showed some level of superficial tissue damage and bleeding at the cortex at the site of the craniectomy, as is commonly observed after FPI of all severities, including mFPI (Fig. 1D).^18,20,21^ Prussian blue staining, which detects past bleeding, showed iron deposits at the superficial cortex of both sham and injured, as is expected from removal of the skull flap.^22^ However, positive staining was not observed within the cortex, corpus callosum, or lateral ventricles in either sham or injured animals in this study (Fig. 1E), which demonstrates that the mFPI parameters used for the study did not cause intracerebral hemorrhage.

### Mild fluid percussion injury does not cause oligodendrocyte death nor does it increase oligodendrocyte progenitor cell numbers in the distal or focal corpus callosum

**FIG. 2.**
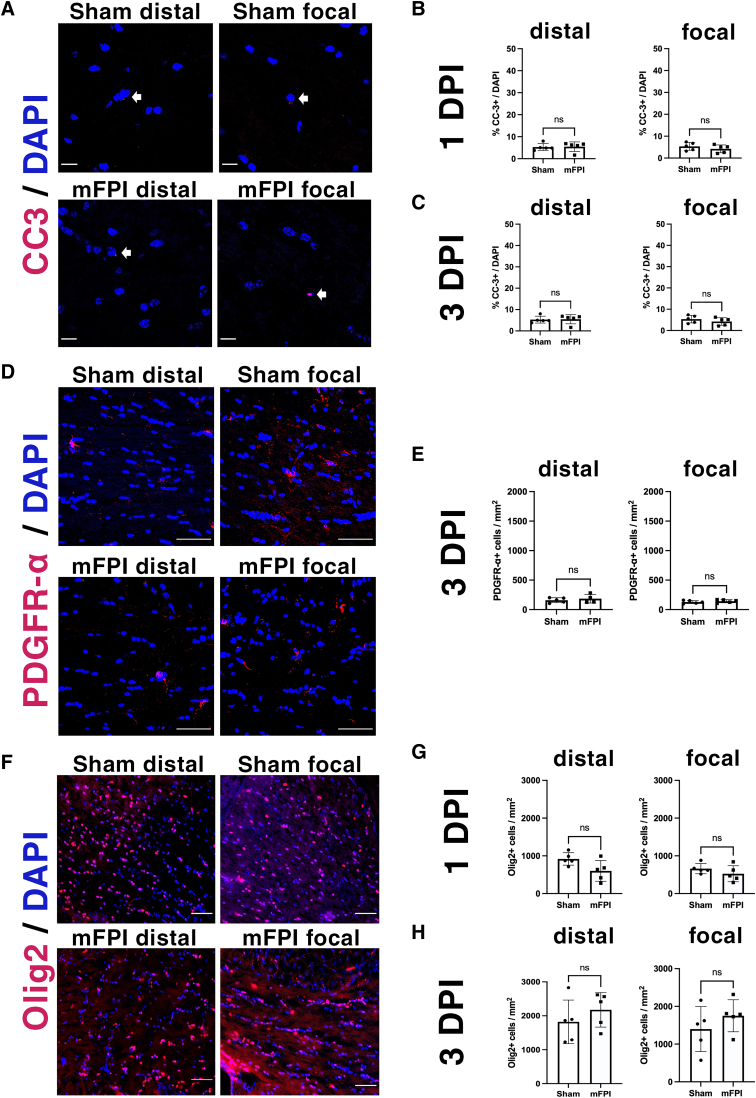
mFPI does not induce apoptosis and has no effect on the overall population of oligodendrocyte lineage cells or OPCs. (**A**) Cleaved caspase-3 immunohistochemistry was used to quantify the number of apoptotic cells after mFPI. Scale bars = 20 μm. (**B,C**) Quantification of the percentage of apoptotic cells in the distal and focal corpus callosum at 1 and 3 DPI. (**D**) Representative images of PDGFR-α immunostaining on the distal and focal corpus callosum in sham and mFPI brains at 3 DPI. Scale bars = 50 μm. (**E**) Number of PDGFR-α^+^ cells in the distal and focal corpus callosum. (**F**) Representative images of immunohistochemistry of Olig2, a transcription factor expressed in all oligodendrocyte lineage cells. Scale bars = 100 μm. (**G,H**) Quantification of the number of Olig2^+^ cells 1 and 3 DPI in the distal and focal corpus callosum. *N* = 5 animals in each group (each data point represents 1 animal). DAPI, 4′,6-diamidino-2-phenylindole; DPI, days post-injury; mFPI, mild fluid percussion injury; Olig2, oligodendrocyte transcription factor 2; OPCs, oligodendrocyte progenitor cells; PDGFR, platelet-derived growth factor receptor.

Moderate-to-severe TBI causes acute mature oligodendrocyte apoptosis^4–6^ whereas mild controlled cortical impact (mCCI) does not.^11^ We observed that, in sham-injured mice, few cells were labeled for cleaved caspase-3 in both the distal and focal corpus callosum (Fig. 2A), indicating a low and steady level of cell death in the non-injured adult mouse brain. At both 1 and 3 DPI, there was no significant difference in the percentage of apoptotic cells between sham and injured animals (Fig. 2B,C). Therefore, unlike moderate-to-severe FPI, mFPI does not cause significant cell death in the corpus callosum.

Recruitment of oligodendrocyte progenitor cells (OPCs) in white matter tracts has been reported within 2 days after moderate FPI.^6^ When immunostained for platelet-derived growth factor receptor (PDGFR)-α, an OPC marker (Fig. 2D), we did not observe any difference in the number of OPCs between the sham and injured groups in either the distal or focal corpus callosum at 3 DPI (Fig. 2D,E). We also compared the total number of oligodendrocyte lineage cells (Olig2^+^) at 1 and 3 DPI (Fig. 2F). No significant difference was observed between sham and mFPI mice (Fig. 2G,H). Therefore, mFPI does not induce cell death nor does it increase the OPC population in the corpus callosum. Moreover, the overall number of oligodendrocyte lineage cells was not altered after mFPI.

### Mild fluid percussion injury causes a decrease in CC1^+^ and BCAS1^+^ oligodendrocytes in the focal, but not distal, corpus callosum

**FIG. 3.**
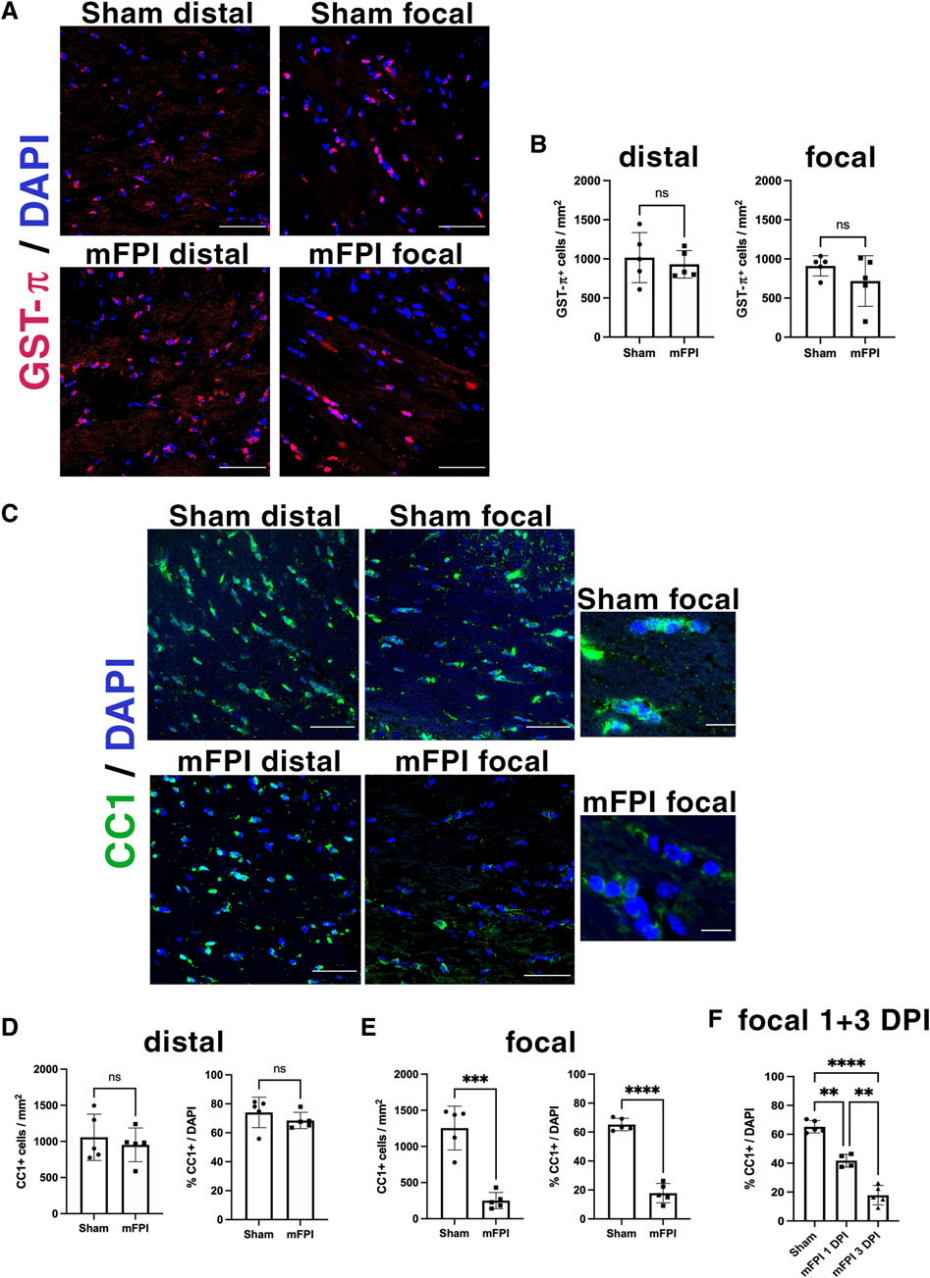
mFPI causes a reduction in the number of CC1-expressing mature oligodendrocytes. (**A**) Representative images of GST-π immunostaining in the distal and focal corpus callosum in sham and mFPI brains at 3 DPI. Scale bars = 50 μm. (**B**) Quantification of the number and percentage of GST-π^+^ cells in the distal and focal corpus callosum at 3 DPI. *N* = 5 animals in each group (each data point represents 1 animal). (**C**) Immunohistochemistry of CC1^+^ mature oligodendrocytes in the corpus callosum of sham and mFPI mice. Scale bars = 50 μm, 10 μm in cropped images. (**D,E**) Quantification of the total number of CC1^+^ cells/mm^2^ and the percentage of CC1^+^ cells in the distal and focal corpus callosum at three DPI. ***p = 0.0001, ****p < 0.0001. (**F**) Quantification of the percentage of CC1^+^ cells in the focal corpus callosum of sham and mFPI animals at 1 and 3 DPI. **p < 0.003, ****p < 0.0001. *N* = 5 animals in each group (each data point represents 1 animal). DAPI, 4′,6-diamidino-2-phenylindole; DPI, days post-injury; GST, glutathione *S*-transferase; mFPI, mild fluid percussion injury.

**FIG. 4.**
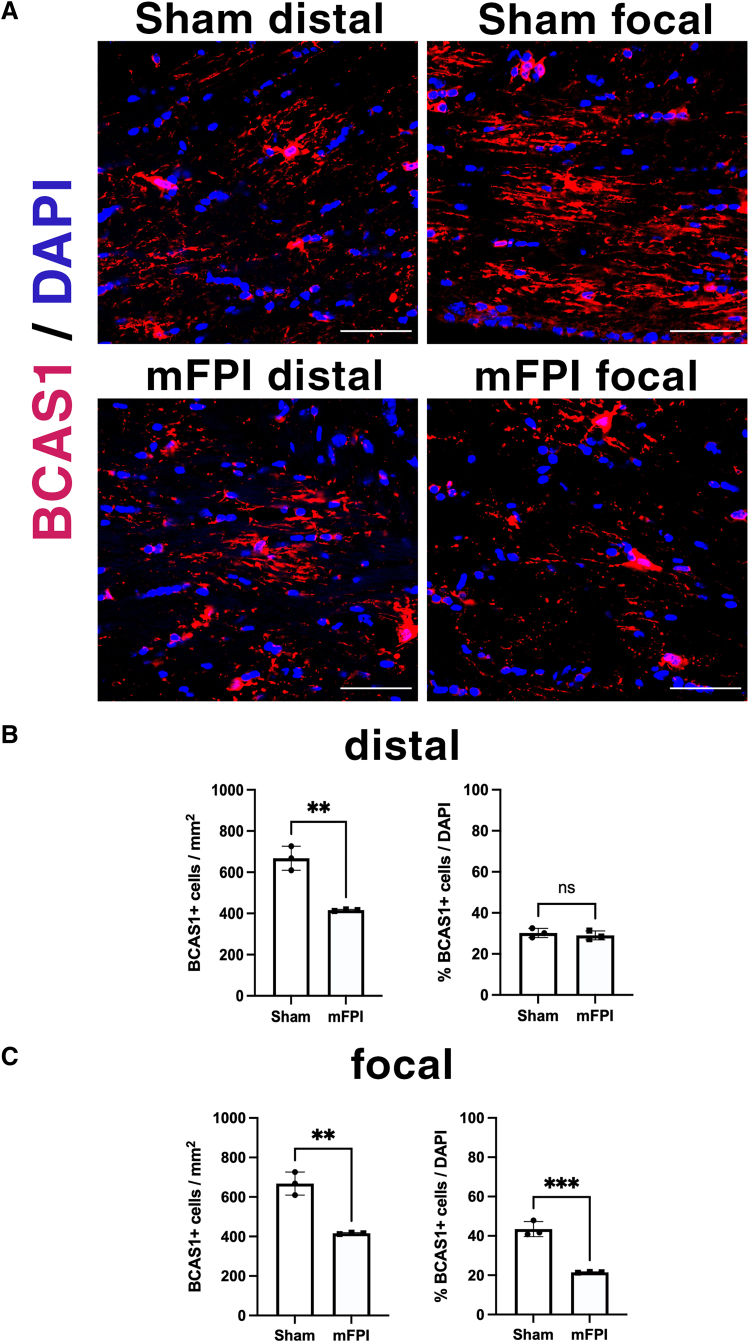
mFPI causes a reduction in the number of BCAS1^+^ actively myelinating mature oligodendrocytes. (**A**) Representative images of BCAS1 expression from the distal and focal corpus callosum of sham and mFPI animals at 3 DPI. (**B,C**) Quantification of the total number and percentage of BCAS1^+^ oligodendrocytes in the distal and focal corpus callosum of sham and mFPI animals. *N* = 3 animals in each group (each data point represents 1 animal). Scale bars = 50 μm. **p = 0.0017, ***p = 0.0006. DAPI, 4′,6-diamidino-2-phenylindole; DPI, days post-injury; mFPI, mild fluid percussion injury.

Next, we investigated the impact of mFPI on the mature oligodendrocyte cell population within the corpus callosum by immunolabeling for glutathione *S*-transferase (GST)-π (Fig. 3A) and CC1 (Fig. 3C). Though both proteins have been used to label mature oligodendrocytes, not all CC1^+^ oligodendrocytes are GST-π^+^ and *vice versa*,^15^ indicating that there are two separate populations of mature oligodendrocytes. At 3 DPI, no difference was observed in GST-π^+^ cell numbers between sham and mFPI mice (Fig. 3B). Interestingly, mFPI elicited a region-specific impact on the CC1^+^ oligodendrocyte population: although no change was observed in the distal region, mFPI caused a significant decrease in the number and the percentage, of CC1^+^ cells in the focal area (Fig. 3D,E). Moreover, mFPI impact was progressive over time between 1 and 3 DPI (Fig. 3F). Breast carcinoma-amplified sequence 1 (BCAS1) protein has been recently identified as a transient maker for a morphologically distinct population of mature oligodendrocytes that are actively forming myelin.^23^ Similar to the impact on CC1^+^ cells, mFPI caused a significant decrease in the percentage of BCAS1^+^ cells at 3 DPI in the focal, but not in the distal, region (Fig. 4B,C). These results show that CC1^+^ and BCAS1^+^ populations of mature oligodendrocytes in the focal corpus callosum are predominantly affected by mFPI (Fig. 2B,C) in the absence of cell death.

### Mild fluid percussion injury causes a reduction in the myelin lipid component in the focal corpus callosum

**FIG. 5.**
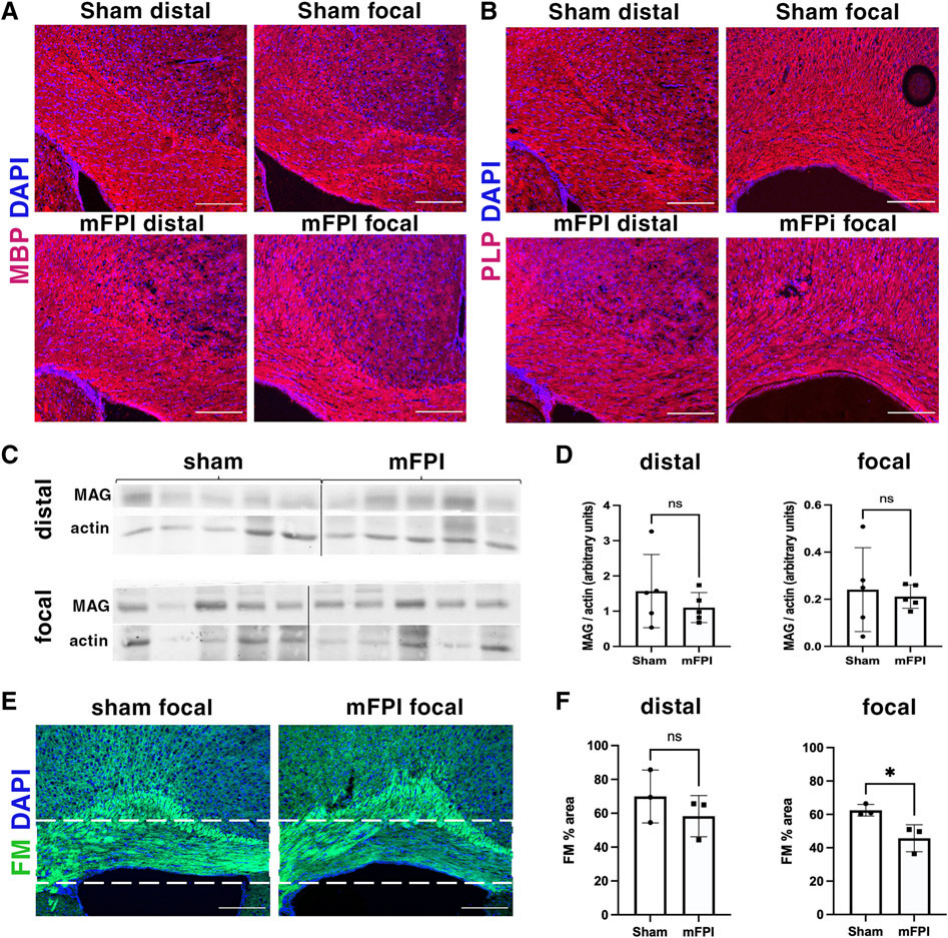
mFPI does not cause a reduction in the expression of myelin proteins, but does reduce the myelin lipid component in the focal corpus callosum at 3 DPI. (**A,B**) Representative images of MBP and PLP immunohistochemistry on sections of the distal and focal corpus callosum of sham and mFPI animals at 3 DPI. (**C**) Immunoblots of MAG from the distal and focal corpus callosum lysates from 5 sham and 5 mFPI-injured mice at 3 DPI. Black vertical lines divide lanes between sham and mFPI animals. (**D**) Quantification of the immunoblot shown in panel C. *N* = 5 animals in each group (each data point represents 1 animal). (**E**) Representative images of FluoroMyelin staining in the focal corpus callosum of sham and injured animals at 3 DPI. White dashed lines outline the corpus callosum. Scale bars in panels A, B, and E = 200 μm. (**F**) Quantification of FluoroMyelin staining, represented as a percentage of area in the distal and focal corpus callosum. *N* = 3 animals in each group (each data point represents 1 animal). *p = 0.0295. DAPI, 4′,6-diamidino-2-phenylindole; DPI, days post-injury; MAG, myelin-associated glycoprotein; MBP, myelin basic protein; mFPI, mild fluid percussion injury; PLP, proteolipid protein.

Myelin-associated proteins myelin basic protein (MBP), proteolipid protein (PLP), and myelin-associated glycoprotein (MAG) are among the most abundant central nervous system myelin proteins. Interestingly, despite the decrease in CC1^+^ and BCAS1^+^ oligodendrocytes, mFPI did not impact protein expression in the corpus callosum (Fig. 5A–D). FluoroMyelin preferentially binds to the lipid components of myelin membrane, making it therefore ideal for visualizing the myelin processes. We observed a significant reduction in the intensity of FluoroMyelin in the focal corpus callosum post-mFPI (Fig. 5E,F). This result suggests that although mFPI does not affect myelin protein expression, it may adversely impact lipid components of the myelin in existing mature oligodendrocytes.

### Mild fluid percussion injury disrupts the node-paranode organization in both the distal and focal corpus callosum

**FIG. 6.**
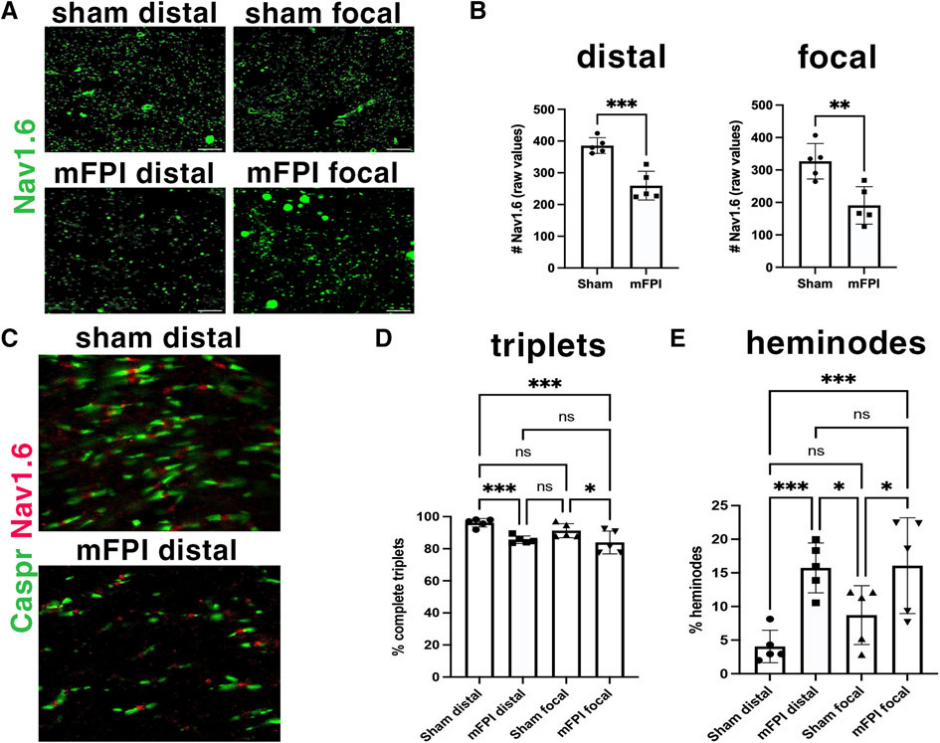
mFPI causes both distal and focal nodal and paranodal abnormalities at 3 DPI. (**A**) Representative images of Nav1.6^+^ nodes of Ranvier in the sham and mFPI corpus callosum (scale bars = 20 μm). (**B**) Quantification of the total Nav1.6^+^ nodes of Ranvier in the distal and focal corpus callosum in sham and mFPI animals. **p = 0.005, ***p = 0.0006. (**C**) Representative images of node-paranode organization visualized by Nav1.6 and Caspr staining in the distal corpus callosum of sham and mFPI animals. (**D,E**) Percentage of complete triplets and heminodes in sham and injured animals in the distal and focal corpus callosum. *N* = 5 animals in each group (each data point represents 1 animal). In panel D, *p = 0.0127, ***p < 0,01. In panel E, *p < 0.05, ***p < 0,0005. DPI, days post-injury; mFPI, mild fluid percussion injury.

Along myelinated axons, each node of Ranvier is flanked by two paranodes opposing oligodendrocyte paranodal loops. Previous studies have shown that mechanical impact caused by TBI disrupts the node-paranode organization, both *in vitro* and *in vivo*.^2,3^ We observed that the total number of Nav1.6^+^ nodes was significantly decreased in both the distal and focal following mFPI at 3 DPI (Fig. 6A and B).

Next, to determine the impact of mFPI on nodal organization, sections were immunostained for Nav1.6 and Caspr, which mark the node and paranode, respectively. In sham mice, complete triplets were clearly identifiable in the distal and focal corpus callosum (Fig. 6C), with few incomplete nodes or “heminodes” associated with only one paranode. mFPI caused a decrease in the number of complete triplets and an inverse increase in the number of heminodes at 3 DPI in both distal and focal regions (Fig. 6D,E). Severity of mFPI impact on node-paranode organization was similar in the distal and focal corpus: There were no significant differences in the number of triplets or heminodes between the distal and focal regions post-mFPI. Therefore, the impact on nodal organization spreads more diffusely, affecting the focal and distal area equally.

**FIG. 7.**
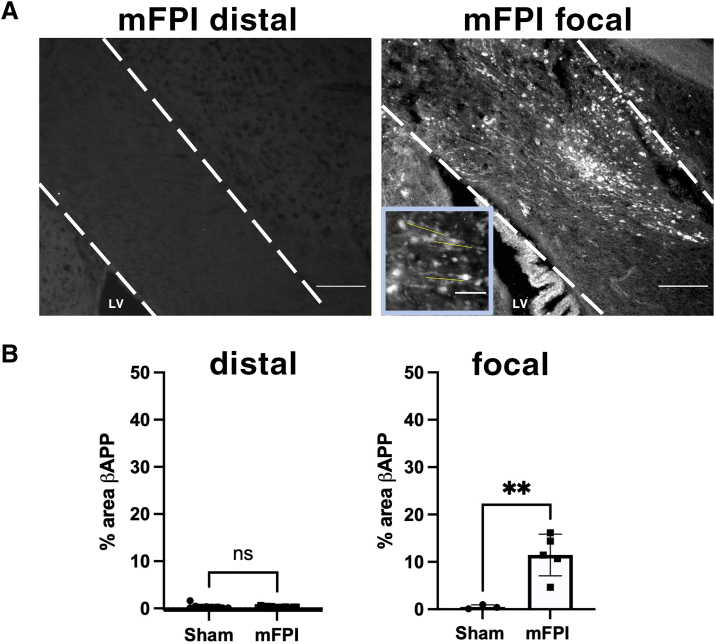
mFPI induced axon damage at 1 DPI in the focal, but not in the distal, corpus callosum. (**A**) Immunohistochemistry was performed on sham and mFPI coronal brain sections for β-amyloid precursor protein (βAPP) as an indicator for axonal damage and impaired transport. White dashed lines outline the corpus callosum, and LV indicates the location of the lateral ventricle. In the focal corpus callosum, damaged axons with accumulation of βAPP were observed throughout the corpus callosum that were most concentrated above the lateral ventricle immediately inferior to the location of impact at the level of the cortex. (**B**) Quantification of βAPP expression as a percentage of area by region. Scale bars = 200 μm in panels A and B, 50 μm in panel C. *N* = 5 animals in each group (each data point represents 1 animal). *p < 0.05, **p = 0.0039, ***p = 0.0005. DPI, days post-injury; mFPI, mild fluid percussion injury.

Loss of Nav1.6 nodes or disruption in node-paranode organization can occur as a consequence of axonal damage. βAPP accumulation is a commonly used indicator of early axonal damage and has been reported in focally injured white matter tracts in rodents after mFPI and mCCI as well as after moderate and severe lateral FPI.^5,9,11,24^ At 1 DPI, in the focal region, the percentage of βAPP^+^ area was significantly increased compared to shams throughout the corpus callosum after mFPI. In contrast, βAPP^+^ axons were rarely found in the distal corpus callosum (Fig. 7). These results suggest that although mFPI-induced early axonal damage may contribute to nodal disruption in the focal region, disruption in the distal region may result from a diffuse injury mechanism independent of extensive axon damage or oligodendrocyte loss.

## Discussion

Expansion of OPCs in white matter tracts during the initial phase of injury has been reported in various experimental TBI models as well as in human patients.^5,6,10,25,26^ OPC proliferation is observed mostly after moderate-to-severe TBI, which also causes oligodendrocyte death.^5,6,25^ This suggests that OPC expansion may occur to replenish the depleted oligodendrocyte pool necessary for remyelination. In our study, we did not observe an expansion of PDGFR-α^+^ OPCs or a change in the oligodendrocyte lineage (Olig2^+^) cell number after mFPI. This may be explained by the lack of dying oligodendrocytes in the area. However, in the absence of oligodendrocyte death, there was a decrease in the number of CC1^+^ as well as BCAS1^+^ mature oligodendrocytes within the oligodendrocyte population, indicating that pre-existing oligodendrocytes may have lost their differentiated phenotype in response to injury. Whether these oligodendrocytes later contribute to remyelination in the absence of OPC expansion is unclear. Though there is no direct evidence that pre-existing oligodendrocytes contribute to remyelination in rodents,^32^ myelin regeneration by pre-existing oligodendrocytes has been suggested in white matter lesions in humans.^33^

One of the key findings in our study is the response of mature oligodendrocytes to mTBI. Mature oligodendrocytes can be marked by the expression of GST-π, CC1, or BCAS1.^23,29,30^ Mature oligodendrocytes can express both CC1 and GST-π, but not all CC1^+^ oligodendrocytes are GSTπ^+^.^10^ In addition, GST-π is expressed in a subpopulation of immature oligodendrocytes^31^ and thus likely marks the oligodendrocytes transitioning from the immature to mature phenotype. We show that although mFPI did not impact GST-π^+^ oligodendrocytes, the injury caused a significant decrease in the CC1^+^ population, which implies an increased vulnerability of more mature oligodendrocytes to mFPI. Our study also shows a significant decrease in BCAS1^+^ oligodendrocytes, which has been shown to represent actively myelinating oligodendrocytes in the adult brain.^23^ Altogether, our results show that although oligodendrocyte populations in the earlier developmental lineage (PDGFR-α^+^, GST-π^+^) are resistant to mTBI, those that are more mature or undergoing active myelination (CC1^+^ or BCAS1^+^) are susceptible to injury.

Expression levels of myelin proteins, such as MBP, PLP, myelin oligodendrocyte glycoprotein, and MAG, are often used to assess the amount of myelin in the white matter tracts. mTBI-induced myelin protein loss has been reported in studies using various experimental models, including closed TBI, ball drop, and blast injury.^13,32,33^ On the other hand, studies using mFPI, including ours (Fig. 5), show that myelin protein expression is unaltered after injury even in areas with evident axonal damage.^9^ A similar finding was observed in a recent study using mild rotational TBI.^14^ The difference in the outcomes is likely attributable to the nature of the injury impacts generated in different experimental models. In our study, however, it is interesting to note that the unaltered myelin protein expression in the focal region was accompanied by a significant decrease in FluoroMyelin staining. FluoroMyelin is a lipophilic dye that selectively detects lipid components of myelin.^34^ This result suggests a selective vulnerability of myelin lipids to mFPI.

On this note, TBI has been shown to cause pronounced alterations in lipid metabolism in the brain as well as systemically.^35–38^ Whether mTBI-induced loss of lipid, but not of protein, components impacts structural integrity of myelin is unknown. It is also possible that the altered lipid-to-protein ratio in myelin may increase its vulnerability to a subsequent injury. Future studies using in-depth ultrastructural and biochemical analyses would be needed to address these interesting questions. Our findings additionally caution against using a single mature oligodendrocyte or myelin marker to ascertain the extent of oligodendrocyte loss or myelin damage, given that multiple markers and analyses may be necessary to fully delineate impact of injury on different populations of mature oligodendrocytes and myelin in white matter tracts.

Disruption in node-paranode organization post-mTBI has been reported. For example, mCCI results in the alteration of node-paranode organization that includes the appearance of asymmetrical paranode pairs and abnormal heminodes 3 days after TBI.^15^ Rotational acceleration mTBI causes loss of Nav1.6^+^ nodes and the appearance of heminodes during the early phase of injury.^39^ The same study reported a similar finding in human TBI brain samples. Further, nodal disruption was not necessarily associated with axonal injury given that the defects were observed in regions with no evidence of β-APP^+^ axonal profiles.^39^ This is in agreement with our findings, in which we observed that mFPI resulted in the loss of Nav1.6^+^ nodes and an increase in heminode appearance in the distal corpus callosum with no detectable βAPP^+^ immunoreactivity.

The molecular mechanisms by which mTBI disrupts node-paranode organization is not clear. It has been shown that mTBI causes a loss of proteins important for the structural organization of nodes and paranodes on myelinated axons even in areas remote from the injury site.^39,40^ A study also reported oligodendrocyte paranodal detachment post-mTBI,^15^ which is likely to disrupt normal node-paranode organization. The diffuse effect of mTBI on node-paranode organization of intact axons is significant given that it is likely to impact neuronal function and white matter connectivity. Accordingly, the co-incidence of paranodal abnormalities and slowed early conduction, followed by conduction failure and loss of functional axons, has been reported after mCCI.^15^

In summary, our data identify a newly found oligodendrocyte phenotype that occurs during the early phase of mTBI. Specifically, we provide evidence suggesting the vulnerability of actively myelinating oligodendrocytes in maintaining the phenotype. We also demonstrate a diffuse impact of mTBI on node-paranode organization, suggesting a possible impact on neuronal connectivity in regions remotely located from the injury site. These findings warrant further studies to explore the impact of mFPI on discrete populations of mature oligodendrocytes and their functions in the adult brain. It is also important to understand the molecular mechanisms underlying the diffuse impact of mTBI on the oligodendrocyte-to-axon interaction that impacts node-paranode organization given that it may provide insight into understanding network dysfunction in the mTBI brain. Further, the acute phase may represent an important time window for therapeutic intervention to prevent further progression of myelin and neuronal damage.

## Supplementary Material

Supplemental data

## Abbreviations Used

βAPP: beta-amyloid precursor protein
BCAS1: breast carcinoma-amplified sequence 1
DPI: days post-injury
FPI: mild fluid percussion injury
GST: glutathione *S*-transferase
H&E: hematoxylin-eosin
MAG: myelin-associated glycoprotein
MBP: myelin basic protein
mCCI: mild controlled cortical impact
mFPI: mild FPI
mTBI: mild TBI
NP-40: Nonidet P-40
OPCs: oligodendrocyte progenitor cells
PDGFR: platelet-derived growth factor receptor
PLP: proteolipid protein
TBI: traumatic brain injury
