# Supplementary material for: Mature and Myelinating Oligodendrocytes Are Specifically Vulnerable to Mild Fluid Percussion Injury in Mice
Source: Neurotrauma Rep. 2023 Jun 29;4(1):433–46. doi: 10.1089/neur.2023.0037 (PMC10331160; doi:10.1089/neur.2023.0037)
Supplement: Supplemental data [file Suppl_Data.docx]

**Supplement: Materials and Methods**

*Mice*

All mice were cared for in accordance with the guidelines of the Institutional Animal Care and Use Committee (IACUC) of Rutgers University. Mice were socially housed with littermates in microisolator cages on IVC racks with a continuous water supply and sterile food pellets and maintained on a standard 12-hour light-dark cycle. All procedures and experiments took place during the light cycle period. C57BL/6 male mice were obtained from Hilltop Labs (Scottdale, PA). Animals underwent craniectomies and experimental brain injury between 3-4 months of age. The total number of animals represented at one or three days post injury or sham are n=20 mFPI mice and n=20 sham injured mice. Tissue from 5 sham and 5 mFPI animals of the 20 in each group were used for western blotting.

*Craniectomies and lateral mild fluid percussion injury*

For both sham and mFPI experiments, animals were deeply anesthetized using 4% isoflurane in 100% oxygen in an induction chamber before being transferred to a stereotaxic frame (Stoelting Co.) and maintained under 2-3% isoflurane in 100% oxygen for the entirety of the procedure. Hair was trimmed as close to the scalp with a manual hair trimmer and sanitized with three alternative swabs of betadine scrub and 70% ethanol. A single midline incision was made from behind the eyes to the base of the neck, and skin flaps were held to the lateral sides of the skull with small bulldog clamps (Roboz Surgical Instrument Co.). Curved forceps were used to remove overlying tissue from the surface of the skull, and a 3 mm circle was drawn on the skull midway between Lambda and Bregma and between the sagittal suture and lateral ridge over the left parietal cortex (0.5 mm from midline), then measured for accuracy before proceeding. Craniectomies of 3 mm in diameter were made using a dental drill (Braintree Scientific Ideal Microdrill) until the skull flap could easily be lifted from the skull surface with fine forceps. After removal of the skull flap, the surface of the brain was examined for any dural breach that would disqualify the animal from the study (and would be subsequently euthanized). The injury hub was created by removing the metal end of a 20g syringe needle (Becton Dickenson) with a razor blade and affixing the plastic cap to the skull using cyanoacrylate glue, which was further applied to the surrounding skull sutures. Dental cement was then applied to the skull surrounding the injury hub and allowed to dry. The hub was filled with 0.9% sterile saline solution to ensure a complete seal between the hub and the skull surface. Animals were then placed in an open cage on top of a heating pad until ambulatory before being returned to a cage with fresh bedding in standard housing. The next day, mice were briefly anesthetized before being subjected to mild lateral fluid percussion injury (mFPI), or sham injury. mFPI was defined by a righting reflex time of 70-270 seconds (2.45±0.988 minutes), similar to what has been reported after lateral mFPI previously Alder et al 2011). Sham injured animals were similarly anesthetized before the experiments and underwent the same experimental procedures but were not subjected to the injury inducing pressure pulse. Righting times for sham animals were consistently lower than one minute for sham-injured animals (0.34±0.13 minutes for sham mice). Injury righting times correlated to pressure pulses of 1.22-1.42 atm (1.33±06 atm or 19.48±0.89 psi). Once ambulatory, animals were returned to their home cage and monitored at least once every 12 hours before reaching their terminal endpoint.

*Tissue Preparation for Immunohistochemistry*

Mice were deeply anesthetized with a ketamine-xylazine cocktail (100 mg/kg and 12 mg/kg, respectively) for transcardial perfusion at one and three post mFPI and sham experiments for the data included in this study (5 sham and 5 mFPI animals at 1 DPI; 5 sham and 5 mFPI animals at 3 DPI). First, animals were perfused with 0.1 M phosphate buffered saline (PBS) supplemented with 10 mM sodium fluoride, then 4% paraformaldehyde and 10 mM sodium fluoride in 0.1 M PBS. Brains were then removed from the skull and immersed in fresh fixative at 4°C overnight. Brains were then briefly rinsed in 0.1 M PBS before cryopreservation in 30% sucrose at 4°C. Tissue was then flash frozen and stored at -80°C before processing. Tissue was mounted in blocks using OTC and frozen, then 14 μm thick serial coronal sections through the corpus callosum were sectioned on a (Leica Biosystems) and mounted onto charged slides (Thermo Fisher Scientific).

*Antibodies*

The following primary antibodies were used for immunohistochemistry and western blot analysis: rabbit anti-Olig2 (1:100 Abcam 109186), anti-mouse APC (CC1, 1:100 EMD Millipore OP80), anti-rabbit cleaved caspase-3 (1:200 Cell Signaling Technology #9661), rabbit anti-β-amyloid precursor protein (βAPP, 1:100 Life Science Technology #51-2700), rabbit anti-GST-π (1:1000 MBL International #312), rabbit anti-platelet derived growth factor receptor-α (PDGFR-α, 1:200 Cell Signaling Technology #3164), rabbit anti-proteolipid protein (PLP, 1:500 Abcam #28486), rabbit anti-myelin basic protein (MBP, 1:500 EMD Millipore MAB980), mouse anti-actin (1:5000 for western blot; Sigma 5441) mouse, anti-myelin associated glycoprotein (MAG, 1:500 for IHC and 1:1000 for western blot; EMD Millipore MAB1567), mouse anti-Caspr (1:500 Neuromab clone K65/35), rabbit anti-Nav1.6 (1:100 Alomone Labs #ASC-009), and breast cancer carcinoma amplified sequence 1 (BCAS1, 1:500 Synaptic Systems 445 003).

*Immunohistochemistry*

Tissue was selected for immunohistochemistry within the focal (interaural positions 4.04-3.56 mm and bregma positions 0.25-0.23 mm) and distal (interaural positions 4.40-4.89 mm and bregma positions 1.09-0.61 mm) corpus callosum. The Allen Brain Atlas was used to confirm the coordinates of the tissue sections used in the study by comparing serially mounted brain sections to the gross anatomy detailed in the Atlas. For all immunostaining procedures, frozen sections were first removed from storage at -80°C, thawed, and dried at room temperature for 15 minutes, then rehydrated with 0.1 M PBS for 10 minutes. Nuclei were counterstained with DAPI (4’,6-diamidino-2-phenylindole; 5 μg/mL; Fisher Scientific) in all procedures. For double immunostaining with CC1 and cleaved caspase-3, tissue sections were permeabilized and blocked with 10% normal donkey serum (NDS) in 0.1% Triton X-100 at room temperature for one hour, then incubated with primary antibody in blocking solution overnight at room temperature. Sections were then rinsed and incubated in secondary antibodies with 3% BSA in PBS for 1.5 hours at room temperature. For double immunostaining of Caspr and Nav1.6, tissue sections were blocked with 0.3 M glycine with 0.1% Triton X-100 in PBS for 30 minutes, followed by a 3-hour block in 25% NDS, 0.3 M glycine, 3% bovine serum albumin, 0.02% sodium azide and 0.2% Triton X-100 in PBS. Sections were then incubated with primary antibodies in 10% NDS, 3% BSA, 0.02% sodium azide and 0.2% Triton X-100 in PBS overnight at 4°C. The incubation period was continued for 3-5 hours at room temperature the next day. Samples were rinsed 3 times in ten-minute intervals with 0.1% Triton X-100 in PBS before incubation in secondary antibodies in 10% NDS, 3% BSA, 0.02% sodium azide and 0.2% Triton X-100 overnight at 4°C. For βAPP single staining, tissue sections were blocked in 25% NDS, 1% BSA, 0.05% sodium azide and 0.4% Triton X-100 in PBS for one hour at room temperature. Sections were then incubated with the primary antibody in 3% BSA in PBS overnight at 4°C. After rinsing 3 times in 10-minute intervals with PBS, sections were incubated in secondary antibodies in 3% BSA in PBS for one hour at room temperature. For Olig2, GST- π, and PDGFR-α single staining, tissue was blocked with 10% NDS in 0.3% Triton X-100 for one hour at room temperature before incubation with the primary antibody at 4°C overnight. Tissue was then rinsed 3 times in 10-minute intervals at room temperature before incubation with secondary antibody at room temperature for one hour. For BCAS1 staining, tissue was permeabilized with 0.25% Triton X-100 in PBS for 30 minutes at room temperature, then rinsed three times in 5-minute intervals. Tissue was blocked with 5% NDS + 1% BSA in PBS for one hour before overnight incubation with the primary antibody with 1% BSA in PBS. The next day, tissue was rinsed three times in 5-minute intervals then incubated with secondary antibodies for one hour at room temperature. Samples were rinsed three times in 10-minute intervals with PBS. For Prussian Blue and FluoroMyelin staining the manufacturer protocols were followed (Sigma Aldrich; Thermo Fisher) All samples were mounted using Fluromount-G (Southern Biotech) with glass coverslips after rinsing and nuclei counterstaining. Images were captured using either a Carl Zeiss MicroImaging LSM 510NLD Meta laser scanning multi-photon confocal microscope, or a Nikon Eclipse TE2000-U microscope equipped with a Hamamatsu Photonics camera using the MicroManager Open Source Imagine Software for camera control.

*Quantification of immunohistochemistry*

All analyses were performed using Fiji opensource software. Only cells residing within the corpus callosum were quantified in all analyses. Imaging parameters for all samples included in each dataset were maintained across sample. For samples double-immunolabeled for CC1 and cleaved caspase-3, single labeled for Olig2, and single labeled for GST-π or PDGFR-α, two images from two non-serial sections were used in the imaging analysis. The percentage of CC1, cleaved caspase-3, Olig2, GST-π and PDGFR-α^+^ cells were calculated based on the number of DAPI labeled nuclei (i.e., the number of total CC1^+^ cells associated with nuclei divided by the total number of nuclei, multiplied by 100). Cells considered CC1, GST-π, PDGFR-α or BCAS1^+^ were categorized as such based on cytoplasmic expression of the protein surrounding a nucleus (labeled by DAPI). Olig2^+^ cells were counted by Olig2 protein expression within the nuclear/perinuclear region of the cell (associated directly with DAPI). Cleaved caspase-3^+^ cells were counted based on the number of cells with cleaved caspase-3 puncta expressed at the cell nucleus. βAPP^+^ axons and Fluromyelin intensity were quantified as a percentage of the total area only within the corpus callosum with βAPP reactivity. All images from sections immunolabeled for βAPP or FluoroMyelin were acquired using the same intensity settings. For analysis, grayscale images were converted to binary, and intensity thresholding was set uniformly across samples. Measurements in Fiji were set to acquire area and area fraction and limited by threshold in order to calculate the percentage of the total area over which βAPP or FluoroMyelin was expressed. The total number of nodes of Ranvier and heminode analyses were performed on images acquired of Nav1.6 and Caspr expression. For each animal, two images from two sections non-serial sections from the distal and focal region were used for analysis. Complete triplets were defined by Nav1.6^+^ nodes of Ranvier flanked on either side by one Caspr segment (the paranode). Heminodes were defined as nodes of Ranvier flanked on only one side by a single Caspr segment. The percentage of heminodes were calculated as the number of heminodes divided by the sum of complete triplets and heminodes, multiplied by 100. The percentage of complete triplets was calculated as the number of complete triplets divided by the sum of complete triplets and heminodes, multiplied by 100. The total number of nodes of Ranvier was calculated using the intensity thresholding and particle analysis functions in Fiji. The intensity threshold and particle size were set uniformly for all images used in the analysis. Particle analysis was used to measure the total number of nodes that fell within the defined size and intensity threshold settings. Any particles out of the size or intensity range were not counted.

*SDS-PAGE and Western Blot analysis*

Sham and injured brains were collected from CO_2_ euthanized animals at 3 DPI. 1 mm coronal slices of the corpus callosum in the distal and focal regions were collected using mouse acrylic brain matrices and lysed in NP-40 lysis buffer (1% NP-40, 1% glycerol, 2.5 mM EGTA, 2 mM EDTA, 1 mM sodium orthovanadate, 1 mM phenylmethylsulphonyl fluoride, 10 mM sodium fluoride, 10 μg/ul aprotinin and 20 μM leupeptin). Tissue was homogenized in lysis buffer using a Dounce homogenizer on ice. Homogenized tissue was collected in 1.5 mL centrifuge tubes and spun for 10 minutes at 13,200 rpm. Total protein in each sample was quantified using the bicinchoninic acid assay (Pierce™ Thermo Fisher Scientific). 25-35 ug of protein was boiled for 10 minutes and separated on 10 or 14% polyacrylamide gels at room temperature. Separated proteins were transferred to polyvinylidene fluoride (PVDF) membranes at 4°C and blocked with 2% non-fat milk (Biorad #1706404XTU) for 1 hour at room temperature. Membranes were then incubated with primary antibodies diluted in TBS (10 mM Tris-HCl and 150 mM NaCl, pH 8.0) with 5% bovine serum albumin overnight at 4°C. Membranes were washed in TBS-T (TBS + 0.1% Tween-20) three times in five-minute intervals before incubation with fluorescent dye conjugated secondary antibodies diluted in 2% non-fat milk with 0.0001% SDS for one hour at room temperature. Membranes were rinsed three times in ten-minute intervals with TBS-T before imaging using the LiCor Odyessy imaging system. Proteins were quantified based on grayscale intensity using ImageJ’s gel quantification features.

*Statistical Analysis*

Statistical analyses were performed using GraphPad Prism Software (version 8). Two-way analysis of variance (ANOVA) with Tukey’s post-hoc analysis was used to determine statistical significance in comparisons of multiple groups, and standard t-test in comparisons of only two groups. P<0.05 was considered significant.
